# Influence of the amount of co-infused amino acids on post-therapeutic potassium levels in peptide receptor radionuclide therapy

**DOI:** 10.1186/s13550-014-0046-2

**Published:** 2014-08-23

**Authors:** Constantin Lapa, Rudolf A Werner, Christina Bluemel, Katharina Lückerath, Andreas Schirbel, Alexander Strate, Andreas K Buck, Ken Herrmann

**Affiliations:** 1 Department of Nuclear Medicine, University Hospital Würzburg, Oberdürrbacher Str. 6, Würzburg 97080, Germany; 2 Institute of Clinical Chemistry, University Hospital Würzburg, Oberdürrbacher Str. 6, Würzburg 97080, Germany

**Keywords:** NET, PRRT, Hyperkalaemia, Arginine, Lysine

## Abstract

**Background:**

Peptide receptor radionuclide therapy (PRRT) is routinely used for advanced or metastasized neuroendocrine tumours (NET). To prevent nephrotoxicity, positively charged amino acids (AA) are co-infused. The aim of this study was to correlate the risk for therapy-related hyperkalaemia with the total amount of AA infused.

**Methods:**

Twenty-two patients undergoing PRRT with standard activities of ^177^Lu-DOTATATE/-TOC were monitored during two following treatment cycles with co-infusion of 75 and 50 g of AA (l-arginine and l-lysine), respectively. Mean serum levels of potassium and other parameters (glomerular filtration rate [GFR], creatinine, blood urea nitrogen [BUN], phosphate, chloride, lactate dehydrogenase) prior to, 4 h and 24 h after AA infusion were compared.

**Results:**

Self-limiting hyperkalaemia (>5.0 mmol/l) resolving after 24 h occurred in 91% (20/22) of patients in both protocols. Potassium levels, BUN, creatinine, GFR, phosphate, chloride and LDH showed a similar range at 4 h after co-infusion of 75 or 50 g of AA, respectively (*p*?>?0.05). Only GFR and creatinine levels at 24 h varied significantly between the two co-infusion protocols (*p*?<?0.05).

**Conclusions:**

Hyperkalaemia is a frequent side effect of AA infusion in PRRT. Varying the dose of co-infused amino acids did not impact on the incidence and severity of hyperkalaemia.

## Background

Peptide receptor radionuclide therapy (PRRT) with radiolabelled somatostatin agonists (^90^Y- or ^177^Lu-DOTATOC/-TATE) is routinely used for advanced neuroendocrine tumours (NET) or other entities such as meningioma that overexpress somatostatin receptor subtype II (SSTR II) [1]-[6]. Whereas the high-energy emitter ^90^Y was first considered more effective, especially in bulky disease [7], some studies reported on higher incidence and severity of side effects like myelosuppression and especially renal toxicity [4],[8]. Since the radiopeptide is reabsorbed at the proximal tubule, subsequent retention may lead to excessive radiation doses and renal failure. In order to prevent significant renal radiopeptide retention, positively charged amino acids (AA) such as l-arginine and l-lysine have been introduced and are used in different protocols for 1 or 2 days [9].

The AA bind to the tubular megalin/cubulin system (which serves as a scavenger system for the reabsorption of AA) and compete with the radiopeptide for reabsorption [10],[11]. The co-administration leads to a significant reduction in the renal absorbed dose [6],[12]. However, side effects like nausea, vomiting and hyperkalaemia have been described [13],[14]. Several authors have reported on the influence of the composition and amount of AA administered on serum potassium levels [11],[13].

The aim of this study was to compare incidence and severity of post-therapeutic hyperkalaemia (>5.0 mmol/l) in patients undergoing PRRT with two different AA infusion regimens administering 50 or 75 g of mixed l-arginine and l-lysine.

## Methods

### Study design and characterization of the patient cohort

Twenty-two consecutive patients (13 males, 9 females) referred for PRRT received 75 g of l-arginine and l-lysine for the first cycle and 50 g of AA for the second cycle, respectively. Mean age was 58?±?14 years (range, 24 to 82 years). The general inclusion and exclusion criteria as defined by *The joint IAEA, EANM and SNMMI practical guidance* were applied [9]. Table 1 gives an overview on patients' characteristics.

**Table 1:** Main baseline features of the patients enrolled in the study

**Patient**	**Age**	**Sex**	**Primary tumour**	**Metastases**	**Previous therapy**
1	76	M	Ileum NET	Liver	Octreotide, interferon alfa
2	62	M	Meningioma	None	None
3	61	M	Pancreatic NET	Local vessel infiltration	Stenting
4	82	F	Pancreatic NET	Liver, bone	Surgery
5	50	M	Rectal NET	Bone	Surgery
6	67	F	Liver NET	LN, muscle, brain	Surgery
7	69	M	Small bowel NET	Liver, lung, LN	None
8	50	F	Pancreatic NET	Liver, LN	Octreotide, everolimus, lanreotide
9	49	M	Small cell lung cancer	LN	CTx
10	73	F	Pancreatic NET	Bone, liver	Surgery
11	58	F	Ileum NET	Liver, LN	Surgery, octreotide, thermoablation
12	57	F	Carcinoid	Lung, bone	Surgery, RCTx
13	31	F	Medullary thyroid cancer	Lung, LN, bone	Surgery, CTx
14	24	M	Ileum NET	Liver	Surgery, TACE, octreotide
15	66	M	Thymus NET	Bone, lung, LN	None
16	60	F	Meningioma	None	Surgery
17	34	M	Paraganglioma	Bone	Surgery, RTx
18	61	M	Small bowel NET	Liver, LN	Surgery, octreotide
19	55	M	Pancreatic NET	Liver, LN	CTx
20	63	M	Pancreatic NET	LN, liver, bone	Surgery
21	68	F	Small bowel NET	Liver, LN	Surgery
22	54	M	Cerebral NET	Bone	CTx

*M* male, *F* female, *NET* neuroendocrine tumour, *LN* lymph nodes, *CTx* chemotherapy, *RCTx* radiochemotherapy, *TACE* transcatheter arterial chemoembolization, *RTx* radiotherapy.

All patients gave written informed consent to the implementation of standard-of-care PRRT. As this study comprised exclusively retrospective analysis of routinely acquired data, our institutional review board (ethics committee of the Medical Faculty of the University of Würzburg) waived the requirement for additional approval. In October 2013, the amount of AA routinely administered was reduced from 75 to 50 g to comply with the latest *joint IAEA, EANM and SNMMI practical guidance*[9].

All patients were observed for two consecutive treatment cycles. Admission occurred one day prior to PRRT itself to allow for adequate hydration. During the first treatment cycle, 1,500 ml of standard solution containing 37.5 g of arginine hydrochloride and 37.5 g of lysine hydrochloride (pH, 7.0) was administered within 4 h (0.5 to 1 h prior to PRRT and for a total of 3 to 3.5 h after therapy; infusion rate, 375 ml/h). For the second cycle, 25 g of each AA in a total of 2,000 ml saline was infused within the same time period (0.5 to 1 h prior to PRRT and for a total of 3 to 3.5 h after therapy; infusion rate, 500 ml/h) according to the most recent practical guidance [9]. The AA solutions were produced in-house according to GMP criteria.

For the first treatment cycle, a mean of 7.5?±?0.7 GBq of ^177^Lu-DOTATATE in 12 ml of normal saline was intravenously infused over 30 min; for the second cycle, 7.6?±?0.3 GBq of ^177^Lu-DOTATOC was administered.

### Preparation of 177Lu-DOTATATE/-TOC

^177^Lu-DOTATATE/-TOC was prepared using a radiotracer synthesis module (Scintomics, Fürstenfeldbruck, Germany) with minor modifications as described before [15]. Briefly, a solution of 200 ?g DOTATATE/-TOC-acetate (ABX, Radeberg, Germany) and 7 mg gentisic acid in 600 ?l sodium acetate buffer (pH?=?4 to 5) was added to a solution of 8 GBq ^177^LuCl_3_ in 0.04 M hydrochloric acid (Isotope Technologies Garching, Garching, Germany) and heated for 30 min at 95°C. The product was diluted with saline and passed through a sterile filter (0.22 ?m) into a sterile vial. Radiochemical purity was determined by gradient high-performance liquid chromatography and thin layer chromatography. Additionally, the product was also tested for pH, sterility and endotoxins.

### Pre- and post-therapeutic blood samples

Blood samples of each patient were drawn to assess standard blood values one day before PRRT and 4 and 24 h after AA infusion. Serum levels of potassium and other parameters (glomerular filtration rate [GFR], creatinine, blood urea nitrogen [BUN], phosphate, chloride, lactate dehydrogenase [LDH]) were compared. Whereas hyperkalaemia is routinely defined by our laboratories as serum K^+^ levels >5.0 mmol/l, severe hyperkalaemia was arbitrarily defined as serum values ?6.0 mmol. Serum potassium levels were measured by indirect ion-sensitive electrode (ISE) (Cobas 8000 system, Roche Diagnostics, Mannheim, Germany). All samples were screened for haemolysis. Absorbances of the diluted serum samples (dilution 1:26) were measured at 570 nm (primary wavelength) and at 600 nm (secondary wavelength), and the haemolysis indices were calculated according to the manufacturer's instruction. Serum samples with haemolysis indices above 90 (equaling 90 mg/dl of free haemoglobin) were considered to be haemolytic and excluded from further evaluation (<5%).

### Analysis and statistics

Statistical analyses were performed using PASW Statistics software (version 22.0; SPSS, Inc. Chicago, IL, USA). Quantitative values were expressed as mean?±?standard deviation and range as appropriate. Comparisons of related metric measurements were performed using Wilcoxon signed rank test.

## Results

### Post-PRRT clinical symptoms

After completion of PRRT, three patients of the 75 g group presented with palpitations, chest pain and/or general discomfort. Evaluation of ECG revealed flattened P waves and high peaked T waves. Two of those three patients also became symptomatic after administration of 50 g AA, whereas the remaining tolerated this regimen without discomfort. In all symptomatic cases, serum potassium levels were ?6.1 mmol/l with peak values of 7.9 mmol/l and required prompt intervention by infusion of 20 IU insulin with 20% glucose in 500 ml saline.

Besides, other side effects being not related to hyperkalaemia were observed in both patient cohorts (e.g. nausea, vomiting and decrease of kidney function).

At baseline, mean K^+^ levels were 4.4?±?0.4 mmol/l (75 g) and 4.3?±?0.4 mmol/l (50 g), respectively. At 4 h, 20/22 subjects (91%) in both groups had serum potassium levels ?5.0 mmol/l with a mean of 5.8?±?0.7 mmol/l (75 g) and 5.7?±?0.8 mmol/l (50 g), respectively. Increases in K^+^ levels were comparable (25% for 75 g, *p*?<?0.001 and 24% for 50 g AA, *p*?<?0.001).

**Figure 1:**
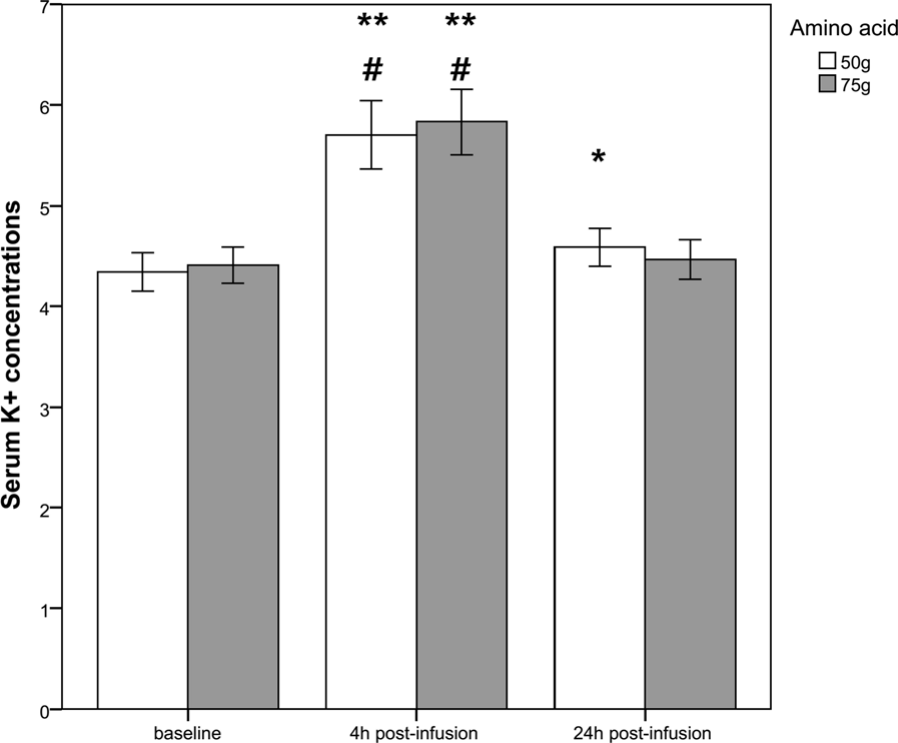
**Serum K**^**+**^**levels.** Serum K^+^ levels at baseline, 4 h post-infusion and 24 h post-infusion (mmol/l; 50 g amino acid vs. 75 g amino acid). **4 h in comparison to baseline (50 g/75 g): *p*?<?0.001; ^#^4 h in comparison to 24 h (50 g/75 g): *p*?<?0.001; *24 h in comparison to baseline (50 g): *p*?<?0.05. Empty bars show serum K^+^ concentrations after administration of 50 g AA, and full bars show serum K^+^ concentrations after administration of 75 g AA.

In both groups, severe hyperkalaemia defined as K^+^ >6.0 mmol/l was observed in 8/22 (36%) patients. After 24 h, K^+^ levels almost returned to baseline (mean, 4.5?±?0.4 mmol/l (75 g AA) vs. mean, 4.6?±?0.4 (50 g AA)) (Figure 1). No significant differences between both AA protocols were found at any time point.

At 4 h post-infusion, all other parameters investigated showed only minor differences. Only glomerular filtration rates (*p*?=?0.011) and creatinine levels at 24 h (*p*?=?0.016) varied significantly between 50 g of AA and 75 g of AA (24 h post-infusion, 75 g vs. 50 g). Table 2 gives an overview of all biochemical parameters.

**Table 2:** Biochemical parameters at baseline, 4 h and 24 h after the beginning of amino acid (AA) infusion

	**Normal range**	**Baseline (75 g AA)**	**Baseline (50 g AA)**	***p*** **value (baseline 75 g vs. 50 g AA)**	**4 h post-infusion (75 g AA)**	**4 h post-infusion (50 g AA)**	***p*** **value (4 h post-infusion 75 g vs. 50 g AA)**	**24 h post-infusion (75 g AA)**	**24 h post-infusion (50 g AA)**	***p*** **value (24 h post-infusion 75 g vs. 50 g AA)**
Potassium (mmol/l)	3.5-5	4.4?±?0.4	4.3?±?0.42	0.491	5.8?±?0.7	5.7?±?0.8	0.338	4.5?±?0.4	4.6?±?0.4	0.243
GFR (ml/min)	>60	89.5?±?27.1	89.4?±?24.6	0.862	85.3?±?24.8	84.0?±?26.2	0.324	84.6?±?26.2	93.2?±?26.9	*0.011*
Creatinine (mg/dl)	<0.95	0.89?±?0.27	0.85?±?0.28	0.444	0.92?±?0.24	0.91?±?0.26	0.952	0.93?±?0.25	0.83?±?0.19	*0.016*
BUN (mg/dl)	10-50	33.28?±?12.86	30.91?±?14.16	0.808	41.88?±?12.23	40.2?±?14.06	0.872	35.62?±?13.81	34.7?±?22.16	0.099
Phosphate (mmol/l)	0.87-1.45	1.08?±?0.19	1.06?±?0.17	0.601	0.85?±?0.16	0.96?±?0.16	0.167	1.16?±?0.25	1.15?±?0.24	0.074
Chloride (mmol/l)	94-110	99.00?±?2.00	109.44?±?27.95	0.180	106.00?±?4.59	109.13?±?2.29	0.102	103.55?±?5.24	107.15?±?2.67	0.157
LDH (U/l)	<250	216.65?±?66.11	205.04?±?57.65	0.811	223.41?±?55.08	257.18?±?128.22	0.953	217.2?±?52.58	247.69?±?82.70	0.674

Values are means?±?SD. *GFR* glomerular filtration rate, *BUN* blood urea nitrogen, *LDH* lactate dehydrogenase, *mmol/l* millimoles per litre, *ml/min* millilitres per minute, *mg/dl* milligrams per decilitre, *U/l* units per litre. Values in italics denote significant difference.

## Discussion

This is the first intra-individual comparison of hyperkalaemia induced by 50 or 75 g of nephroprotective amino acids (Arg-Lys) in patients with neuroendocrine tumours undergoing PRRT. Incidence and severity of hyperkalaemia (>5.0 mmol/l) were comparable for both protocols.

In PRRT, AA-induced hyperkalaemia is believed to result from the ketogenic characteristics of lysine, lowering intracellular pH and thereby causing an outwardly directed K^+^ flux [14]. Various publications have previously reported on AA-induced increase of serum potassium levels. For example, Barone and colleagues found just marginal increase in K^+^ levels in patients treated with a mixture of arginine and lysine totaling 50 g [11]. However, only six patients were included in this study and blood samples were drawn at an early time point (2.5 h after the start of infusion). Recently, a group from Switzerland reported on hyperkalaemia after administration of 25 g lysine and 25 g arginine. In their cohort, serum potassium levels increased significantly 4 h after the beginning of AA infusion [16]. Last, Rolleman et al. reported on even more pronounced K^+^ increases induced by 75 g of lysine, warning of AA-induced hyperkalaemia. In our cohort, we observed higher values of potassium than previously reported [13]. The reason for this observation is not fully understood. In order to rule out spurious hyperkalaemia, blood haemolysis indices were calculated. One possible explanation might be the pH value of the amino acid solution administered, which in our department is 7.0. Serum acidosis is known to be associated with potassium movement from the cellular space into the extracellular fluid in order to buffer excess hydrogen ions. The rather high volume infusion of 1,500 ml of AA might affect serum pH and thereby subsequently influence serum potassium. The total amount of AA solution administered did not influence incidence or severity of hyperkalaemia. Therefore, our data hint at a major contribution of the pH of the AA solution which was the same in all cycles.

Both infusion protocols were equally well tolerated. Most of the patients experienced asymptomatic rises in K^+^, and symptoms resolved quickly within 24 h without any further intervention. Three patients became symptomatic including palpitations, chest pain and general discomfort. Two of those three patients suffered from hyperkalaemia-related side effects regardless of the amount of AA infused, whereas the remaining subject only experienced discomfort with 75 g of AA. Interestingly, this subject had lower K^+^ values after administration of 50 g AA (6.1 mmol/l) as compared to 75 g AA (7.0 mmol/l). In all, treatment with insulin and glucose was highly effective and symptoms were relieved quickly.

Changes in other serum parameters included a non-significant decrease in phosphate, increase in BUN or changes in chloride which were also not significant, consistent with studies published before [16]. Also, no significant changes in kidney function were observed. According to our study, only a transient increase of serum creatinine after start of the AA infusion has been reported [11] and might be associated with higher doses of AA due to non-specific tubular cell blockade. Resolution was observed after 24 h. In short, hyperkalaemia as an adverse event to a potentially life-threatening degree has to be kept in mind regardless of the AA protocol used for PRRT.

The following limitations have to be considered: firstly, the retrospective study design and the low patient number may be associated with a significant sample bias. Secondly, this study does not allow any conclusions regarding the nephroprotective effect of the different AA amounts. Therefore, future prospective studies with larger patient numbers need to be performed, potentially also investigating the long-term effectiveness and safety of AA co-infusion protocols with even lower AA amounts. However, to the best of our knowledge, this is the first report on an intra-individual comparison of two different AA protocols.

## Conclusions

Transient hyperkalaemia is a frequent side effect of AA co-infusion during PRRT. In contrast to previous reports, incidence and severity of hyperkalaemia do not significantly vary between different amounts of AA administered.

## Competing interests

The authors declare that there are no competing interests.

## Authors¿ contributions

KL, AnS, AKB and KH conceived and designed the study. CB and AlS acquired the data. CL and RAW analyzed and interpreted the data. CL, RAW, CB and AlS were involved in the drafting of the manuscript, and KL, AnS, AKB and KH revised it critically for important intellectual content. All authors gave final approval of the version to be published and agree to be accountable for all aspects of the work in ensuring that questions related to the accuracy or integrity of any part of the work are appropriately investigated and resolved.
